# Defects in autophagosome-lysosome fusion underlie Vici syndrome, a neurodevelopmental disorder with multisystem involvement

**DOI:** 10.1038/s41598-017-02840-8

**Published:** 2017-06-14

**Authors:** Ikumi Hori, Takanobu Otomo, Mitsuko Nakashima, Fuyuki Miya, Yutaka Negishi, Hideaki Shiraishi, Yutaka Nonoda, Shinichi Magara, Jun Tohyama, Nobuhiko Okamoto, Takeshi Kumagai, Konomi Shimoda, Yoshiya Yukitake, Daigo Kajikawa, Tomohiro Morio, Ayako Hattori, Motoo Nakagawa, Naoki Ando, Ichizo Nishino, Mitsuhiro Kato, Tatsuhiko Tsunoda, Hirotomo Saitsu, Yonehiro Kanemura, Mami Yamasaki, Kenjiro Kosaki, Naomichi Matsumoto, Tamotsu Yoshimori, Shinji Saitoh

**Affiliations:** 1 Department of Pediatrics and Neonatology, Nagoya City University Graduate School of Medical Sciences, Nagoya, 467-8601 Japan; 2 Department of Genetics, Osaka University Graduate School of Medicine, Osaka, 565-0871 Japan; 3 Research Center for Autophagy, Osaka University Graduate School of Medicine, Osaka, 565-0871 Japan; 4 Department of Human Genetics, Yokohama City University Graduate School of Medicine, Yokohama, 236-0004 Japan; 5 Department of Medical Science Mathematics, Medical Research Institute, Tokyo Medical and Dental University, Tokyo, 113-8510 Japan; 6 Laboratory for Medical Science Mathematics, RIKEN Center for Integrative Medical Sciences, Yokohama, 230-0045 Japan; 7 Department of Pediatrics, Hokkaido University Graduate School of Medicine, Sapporo, 060-8638 Japan; 8 Department of Pediatrics, Kitasato University School of Medicine, Sagamihara, 252-0373 Japan; 9 Department of Pediatrics, Epilepsy Center, Nishi-Niigata Chuo National Hospital, Niigata, 950-2085 Japan; 10 Department of Medical Genetics, Osaka Medical Center and Research Institute for Maternal and Child Health, Osaka, 594-1101 Japan; 11 Department of Pediatrics, Wakayama Medical University, Wakayama, 641-8509 Japan; 12 Department of Pediatrics, Graduate School of Medicine, The University of Tokyo, Tokyo, 113-8655 Japan; 13 Department of Neonatology, Ibaraki Children’s Hospital, Mito, 311-4145 Japan; 14 Department of Child Health, Faculty of Medicine, Tsukuba University, Tsukuba, 305-8576 Japan; 15 Department of Pediatrics, Faculty of Medicine, Tokyo Medical and Dental University, Tokyo, 113-8519 Japan; 16 Department of Radiology, Nagoya City University Graduate School of Medical Sciences, Nagoya, 467-8601 Japan; 17 Department of Neuromuscular Research, National Institute of Neuroscience, National Center of Neurology and Psychiatry, Tokyo, 187-8551 Japan; 18 Department of Pediatrics, Showa University School of Medicine, Tokyo, 142-8666 Japan; 19 Department of Biochemistry, Hamamatsu University School of Medicine, Hamamatsu, 431-3192 Japan; 20 Division of Regenerative Medicine, Institute for Clinical Research, Osaka National Hospital, National Hospital Organization, Osaka, 540-0006 Japan; 21 Department of Neurosurgery, Osaka National Hospital, National Hospital Organization, Osaka, 540-0006 Japan; 22 Department of Neurosurgery, Takatsuki General Hospital, Osaka, 569-1192 Japan; 23 Center for Medical Genetics, Keio University School of Medicine, Tokyo, 160-8582 Japan

## Abstract

Vici syndrome (VICIS) is a rare, autosomal recessive neurodevelopmental disorder with multisystem involvement characterized by agenesis of the corpus callosum, cataracts, cardiomyopathy, combined immunodeficiency, developmental delay, and hypopigmentation. Mutations in *EPG5*, a gene that encodes a key autophagy regulator, have been shown to cause VICIS, however, the precise pathomechanism underlying VICIS is yet to be clarified. Here, we describe detailed clinical (including brain MRI and muscle biopsy) and genetic features of nine Japanese patients with VICIS. Genetic dissection of these nine patients from seven families identified 14 causative mutations in *EPG5*. These included five nonsense, two frameshift, three splicing, one missense, and one multi-exon deletion mutations, and two initiation codon variants. Furthermore, cultured skin fibroblasts (SFs) from two affected patients demonstrated partial autophagic dysfunction. To investigate the function of *EPG5*, siRNA based *EPG5* knock-down, and CRISPR/Cas9 mediated *EPG5* knock-out HeLa cells were generated. *EPG5*-depleted cells exhibited a complete block of autophagic flux caused by defective autophagosome-lysosome fusion. Unexpectedly, endocytic degradation was normal in both VICIS SFs and *EPG5* depleted cells, suggesting that *EPG5* function is limited to the regulation of autophagosome-lysosome fusion.

## Introduction

Vici syndrome (VICIS, OMIM #242840) is a rare, autosomal recessive neurodevelopmental disorder characterized by agenesis of the corpus callosum (ACC), cataracts, cardiomyopathy, combined immunodeficiency, developmental delay, and hypopigmentation^1^. Since first described in 1988 by Dionisi Vici *et al*.^1^, multiple case reports that describe multisystem involvement suggest that a fundamental molecular defect in cell physiology underlies the disorder^2–11^. Mutations in the *EPG5* gene on chromosome 18q12.3 were recently identified to cause VICIS^12–19^. *EPG5* is the human homolog of the metazoan-specific autophagy gene *epg-5*, encoding a key autophagy regulator (ectopic P-granules autophagy protein 5)^20^. Autophagy is an evolutionary conserved lysosomal degradation process essential for cell homeostasis^21–23^. The function of *epg-5* was initially reported in *C. elegans*, where it is required for the formation of degradative autolysosomes^20^. Using *Epg5* knock-out (KO) mice, Zhao *et al*.^24, 25^ demonstrated that *Epg5* was involved in the maturation of autophagosomes into degradative autolysosomes, as well as endocytic degradation and recycling. However, the precise mechanism of *Epg5* remains to be elucidated, and its role in human cells has not been intensively studied.

Here, we studied nine patients with VICIS from seven families and identified 14 causative mutations in *EPG5*. Skin fibroblasts (SFs) from VICIS patients and *EPG5* knock-down (KD) and KO HeLa cells demonstrated normal endocytic function, but impaired autophagic function due to defects in autophagosome-lysosome fusion.

## Results

### Clinical features

We genetically identified nine patients (three males and six females) with VICIS, including two pairs of siblings, from seven Japanese families. Table 1 provides a summary of the clinical features of our nine patients and reported patients in the literature^1–19^. Typical clinical features are illustrated in Supplementary Fig. S1. No consanguinity was noted in any of the families. All patients had a normal gestation and delivery, with normal birth weight, length and head circumference. All patients presented with developmental delay, hypotonia, and recurrent infections. Developmental delay was profound and no patients acquired meaningful words. Most patients developed progressive microcephaly and failed to thrive (Supplementary Table S1). Two patients died; patient 2.1 at the age of 14 years due to respiratory arrest, and patient 5.1 at the age of one year due to cardiomyopathy. Cardiomyopathy and cataracts, both initially described as principle features in VICIS^1^, were notably uncommon (3/9 cases) or absent (0/9 cases), respectively, in these patients. Most of the patients (8/9) showed intractable epilepsy with epileptic spasms, tonic and myoclonic seizure types, and one patient exhibiting symptomatic West syndrome. Seizures started at the median age of one year and two months. Although cataracts were not present in any patient, abnormal ophthalmologic findings were identified in seven patients; two with optic disc pallor, two with erratic eye movement and five with nystagmus. Complete ACC was observed in all patients. Additional CNS abnormalities included paucity of white matter, irregularity of the ventricular wall, ventricular dilation, delayed myelination, pontine hypoplasia, cerebellar hypoplasia and cerebral atrophy while typical Probst bundle was not detected in any patients (Supplementary Fig. S2). Increased serum aspartate transaminase (AST) and alanine transaminase (ALT) was shown in all patients and increased serum creatine kinase was present in seven patients. Muscle biopsies were performed in four patients and showed only mild myopathic changes including fiber-type disproportion with type 2 atrophy (Supplementary Fig. S3a–f). Electron microscopy was performed on one patient biopsy (2.1) and revealed only slight abnormalities of autophagosome vacuoles (Supplementary Fig. S3g and h).

**Table 1:** Clinical features of nine patients with Vici syndrome in current study and summary of previous reports.

Family	1		2		3	4	5	6	7	Summary of our 9 patients	Summary of previously reported 67 patients^1–19^	Total summary of all 76 patients
Subject	1.1	1.2	2.1	2.2	3.1	4.1	5.1	6.1	7.1			
Sex	F	F	M	F	M	M	F	F	F	3 male	13 male	16 male
										6 female	12 female	18 female
											42 NA	42 NA
Status	Alive	Alive	Dead^a^	Alive	Alive	Alive	Dead^b^	Alive	Alive	7 Alive	14 Alive	21 Alive
	7y	2y	14y	15y	5y	7y	1y	4y	2y	2 Dead	20 Dead	22 Dead
											33 NA	33NA
Developmental delay	+	+	+	+	+	+	+	+	+	9/9 (100%)	58/58 (100%)	67/67 (100%)
Hypotonia	+	+	+	+	+	+	+	+	+	9/9 (100%)	25/25 (100%)	34/34 (100%)
Hypopigmentation	+	+	−	−	+	+	+	+	+	7/9 (78%)	65/67 (97%)	72/76 (95%)
Cardiomyopathy	+	−	−	−	+	−	+	−	−	3/9 (33%)	49/59 (83%)	52/68 (76%)
Seizures	+	+	+	+	+	+	+	+	−	8/9 (89%)	17/29 (59%)	25/38 (66%)
Seizure onset	1y4m	2m	1y10m	5m	1y5m	1y5m	5m	1y0m				
Seizure type	Spasm	Spasm	Tonic	Tonic	West	Tonic	Spasm	Myoclonic tonic				
Ophthalmologic finding												
Cataracts	−	−	−	−	−	−	−	−	−	0/9 (0%)	48/63 (76%)	48/72 (67%)
Optic disc pallor or atrophy	−	−	−	−	+	−	−	+	−	2/9 (22%)	9/9 (100%)	11/18 (61%)
Erratic eye movement	−	−	−	−	−	+	+	−	−	2/9 (22%)	NA	2/9 (22%)
Nystagmus	−	−	+	+	+	−	+	+	−	5/9 (56%)	11/21 (52%)	16/30 (53%)
Hypopigmented iris	−	−	−	−	−	−	−	−	+	1/9 (11%)	8/10 (80%)	9/19 (47%)
Recurrent infection	+	+	+	+	+	+	+	+	+	9/9 (100%)	62/64 (97%)	71/73 (97%)
Microcephaly	+	−	+	+	+	+	+	−	+	7/9 (78%)	43/48 (90%)	50/57 (88%)
High-arched palate	+	+	+	+	+	+	+	+	+	9/9 (100%)	15/24 (63%)	24/33 (73%)
Central nervous system anomalies												
ACC	+	+	+	+	+	+	+	+	+	9/9 (100%)	65/65 (100%)	74/74 (100%)
Paucity of white matter	+	−	+	+	+	−	−	+	NA	5/8 (63%)	5/6 (83%)	10/14 (71%)
Irregularity of the ventricular wall	+	−	+	+	+	+	+	+	NA	7/8 (88%)	NA	7/8 (88%)
Ventricular dilation	+	+	+	+	+	−	+	+	+	8/9 (89%)	8/11 (73%)	16/20 (80%)
Delayed myelination	−	−	−	−	+	+	−	−	NA	2/8 (25%)	4/6 (67%)	6/14 (43%)
Pontine hypoplasia	+	+	+	+	+	+	+	+	+	9/9 (100%)	5/9 (56%)	14/18 (78%)
Hypoplasia of the medulla oblongata	−	−	−	−	−	−	−	−	−	0/9 (0%)	NA	0/9 (0%)
Cerebellar hypoplasia	−	−	+	+	+	−	−	+	NA	4/8 (50%)	13/16 (81%)	17/24 (71%)
Cerebral atrophy	+	±	+	+	+	+	−	+	NA	6/8 (75%)	5/9 (56%)	11/17 (65%)
Probst bundles	−	±	−	−	−	−	−	−	NA	0/8 (0%)	1/4 (25%)	1/12 (8%)
Elevated CK	+	+	+	+	−	+	−	+	+	7/9 (78%)	11/13 (85%)	18/22 (82%)
Elevated AST/ALT	+	+	+	+	+	+	+	+	+	9/9 (100%)	12/13 (92%)	21/22 (95%)
Abnormal immunology	−	−	−	−	+	+	−	−	−	2/9 (22%)	15/23 (65%)	17/32 (53%)
Abnormal muscle biopsy	−	NA	−	−	NA	−	NA	NA	NA	0/4 (0%)	20/21 (95%)	20/25 (80%)

F = female, M = male, ACC = agenesis of the corpus callosum, NA = not available, CK = creatine kinase, AST = aspartate transaminase, ALT = alanine transaminase, ^a^Patient 2.1 died at the age of 14 years due to respiratory arrest. ^b^Patient 5.1 died at the age of one year due to cardiomyopathy.

### Molecular genetic analyses

**Figure 1:**
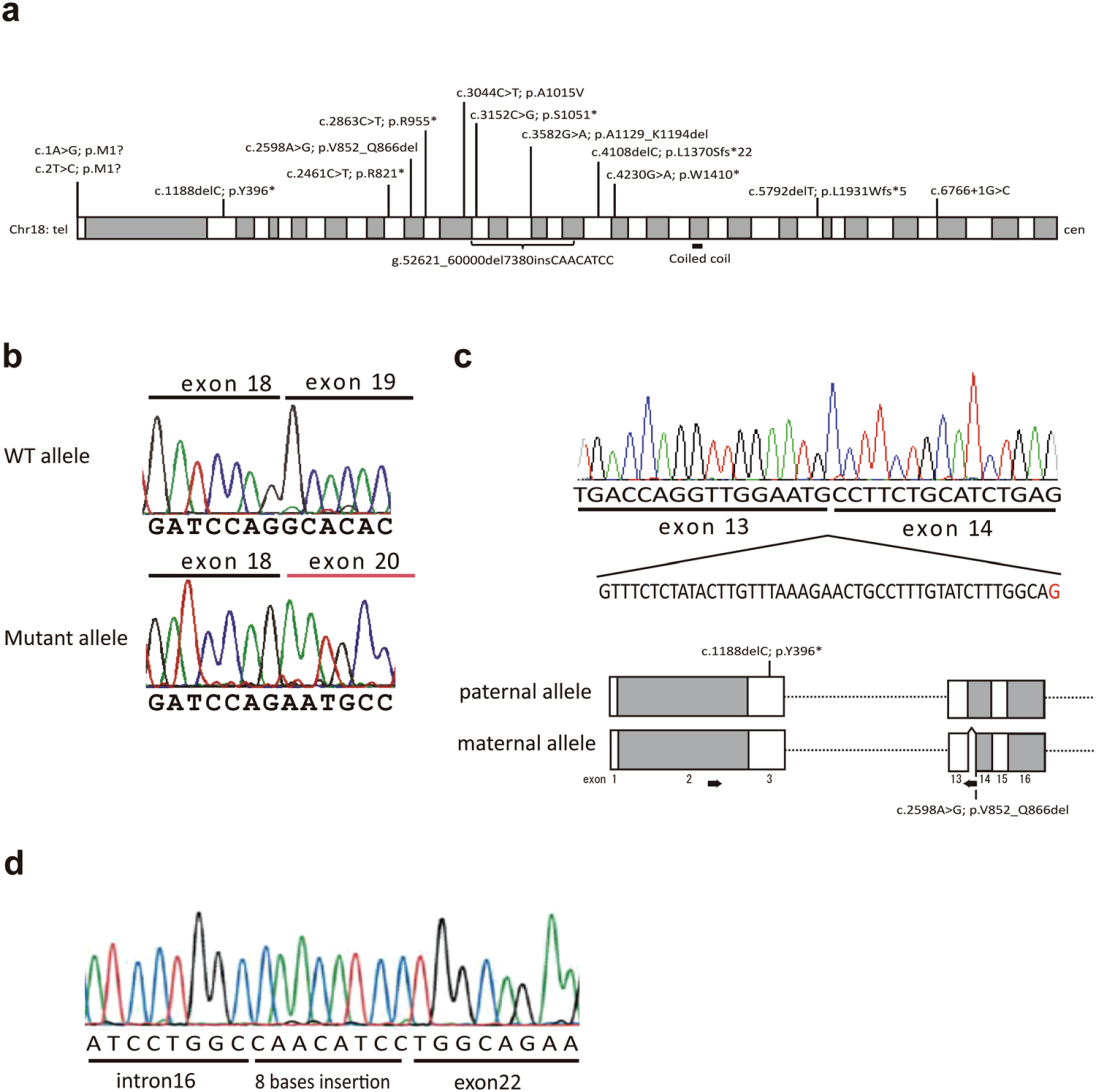
*EPG5* mutations identified in nine Vici syndrome patients. (**a**) Schematic of the *EPG5* gene located in 18q12.3 illustrating mutation locations. White and gray rectangles represent exons (1 through 44). (**b**) Aberrant splicing caused by the synonymous mutation in patient 3.1 (c.3582 G > A) identified the removal of exon 19. WT, wild type. (**c**) Aberrant splicing caused by the synonymous mutation in patient 5.1 (c.2598 A > G). *Above*; RT-PCR identified a 45-bp in-frame deletion in exon 14 induced by a cryptic splicing acceptor site created by 2598 A > G (shown in red). The mother of patient 5.1 also had this mutation. *Below*; Confirmation of compound heterozygosity. The paternal allele was amplified by PCR using a reverse primer that binds to the region deleted in the maternal allele. The paternal PCR product contained the c.1188delC mutation, indicating that patient 5.1 was a compound heterozygote. The location of the mutation in patient 5.1 is shown. The arrows represent PCR primers. White and gray rectangles represent exons. (**d**) A multi-exon deletion identified in patient 7.1. Cloning and sequencing of the genomic breakpoint and flanking regions revealed an 8-bp insertion of unknown origin at the breakpoint.

Of the nine patients with VICIS, eight patients from six families were identified by whole exome sequencing, while one patient (patient 7.1) was identified by the target panel analysis. Compound heterozygous mutations in *EPG5* were identified in all patients (Table 2). The 14 *EPG5* mutations identified were all novel and comprised five nonsense, two frameshift, three splicing, one missense, one multi-exon deletion, and two initiation codon variants (Fig. 1a). The single missense mutation (p.Ala1015Val) was predicted to be damaging by SIFT (score 0.00), PolyPhen-2 (HumVar score 0.656) and MutationTaster (probability value 0.999). One splicing mutation (c.6766 + 1 G > C), found in patient 6.1, was located at the canonical +1 splice site. The two remaining splicing mutations (c.3582 G > A and c.2598 A > G) found in patients 3.1 and 5.1, respectively, were confirmed as pathogenic by reverse transcriptase-polymerase chain reaction (RT-PCR). The c.3582 G > A mutation was located in the last codon of exon 19. RT-PCR showed that removal of exon 19 resulted in an in-frame deletion of 66 amino acid residues (p.Ala1129_Lys1194del) (Fig. 1b). The c.2598 A > G mutation generated a cryptic splicing site, which introduced a 45-bp in-frame deletion of 15 amino acid residues (p.Val852_Gln866del) (Fig. 1c). A multi-exon deletion of exons 17 to 21 in entirety and the 5’-end of exon 22 was identified in patient 7.1. Cloning the breakpoint identified a 7,380 bp deletion and an 8 bp insertion (Fig. 1d). Both parents from five families (Family1–4, 6) were revealed as carriers. One of the mutations in patient 5.1 arose *de novo* (c.1188delC; p.Tyr396*) on the paternal allele, while the other mutation was maternally inherited (c.2598 A > G). We verified the paternity in family 5 by confirming the segregation of SNPs. The parents’ samples from patient 7.1 were not available.

**Table 2:** Mutations identified in the nine Vici syndrome patients.

Family	Subject	Allele 1			Allele 2		
		Nucleotide	Amino acid	Origin	Nucleotide	Amino acid	Origin
1	1.1	c.2 T > C	p.Met1?	M	c.5792delT	p.Leu1931Trpfs*5	P
	1.2	c.2 T > C	p.Met1?	M	c.5792delT	p.Leu1931Trpfs*5	P
2	2.1	c.3152 C > G	p.Ser1051*	P	c.4230 G > A	p.Trp1410*	M
	2.2	c.3152 C > G	p.Ser1051*	P	c.4230 G > A	p.Trp1410*	M
3	3.1	c.2461 C > T	p.Arg821*	P	c.3582 G > A	splicing (p.Ala1129_Lys1194del)	M
4	4.1	c.1 A > G	p.Met1?	M	c.4108delC	p.Leu1370Serfs*22	P
5	5.1	c.1188delC	p.Tyr396*	P (*de novo*)	c.2598 A > G	splicing (p.Val852_Gln866del)	M
6	6.1	c.3044 C > T	p.Ala1015Val	P	c.6766 + 1 G > C	—	M
7	7.1	c.2863 C > T	p.Arg955*	NT	multi-exon deletion (g.52621_60000del7380insCAACATCC)		NT

The numbering of the mutations and alterations is relative to NM_020964.2 (gene) and NP_066015.2 (protein), respectively. With regard to multi-exon deletions, the variant’s description is relative to genomic sequence records for NG_042838.1. Synonymous mutations in patient 3.1 and patient 5.1 were found to cause an in-frame deletion on the basis of cDNA sequence data derived from affected individuals. ?protein has not been analysed, an effect is expected but difficult to predict, *=the protein coding sequence ends at a translation termination codon, P = paternal, M = maternal, NT = not tested.

### Accumulation of autophagosomes in VICIS SFs

**Figure 2:**
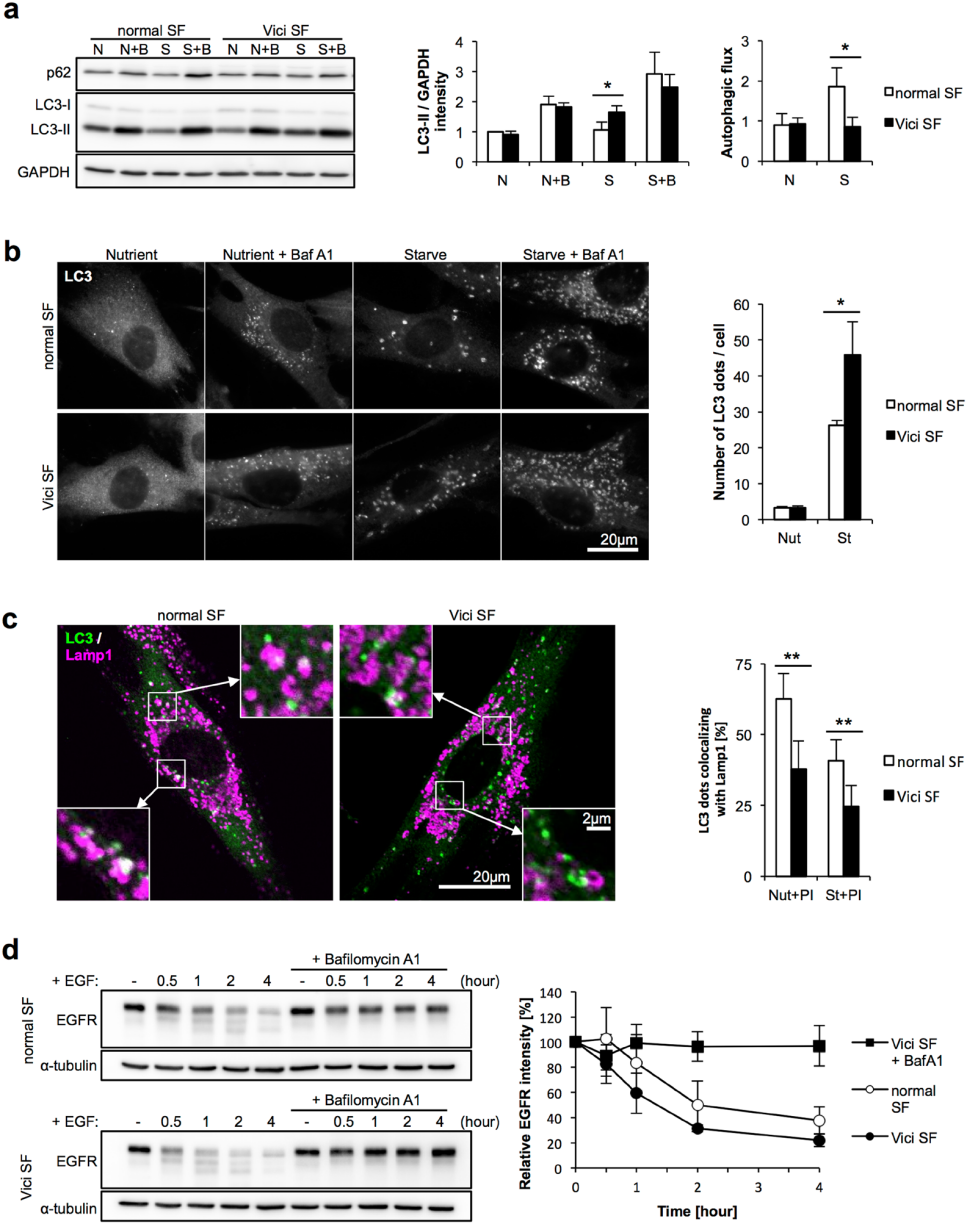
Mild autophagic impairment in VICIS SFs. (**a**) Normal and VICIS SFs were incubated in nutrient-rich or starved conditions with/without BafA1 for 6 hours. A representative western blot of LC3 and p62, and quantitative analysis of band intensities are shown. LC3 intensities were standardized by GAPDH intensities, and values relative to that of normal cells in nutrient-rich conditions without BafA1 are shown. Autophagic flux was calculated by subtracting values for BafA1 untreated samples from treated samples in each condition. Upon starvation, VICIS SFs exhibited mild accumulation of LC3-II and reduced autophagic flux. p62 is another substrate which is degraded by autophagy, but it showed little variation in expression. (**b**) SFs were incubated in nutrient-rich or starved conditions with/without BafA1 for 2 hours, followed by LC3 immunostaining. Representative images and quantified numbers of punctate LC3 staining per cell are shown. Under starved conditions, LC3 punctate staining was higher in VICIS SFs compared with normal SFs, which is consistent with the increased intensity of the LC3-II band in VICIS SFs under the starved conditions shown in (**a**). (**c**) SFs were incubated in nutrient-rich or starved conditions with/without protease inhibitors for 2 hours, followed by LC3 and Lamp1 immunostaining. Representative images and calculated percentages of punctate LC3 staining colocalized with Lamp1 are shown. Decreased colocalization between LC3 and Lamp1 staining in VICIS SFs indicates reduced autophagosome-lysosome fusion. (**d**) Conventional endocytic pathway activity assessed by EGFR degradation was normal in VICIS SFs. N or Nut, nutrient-rich conditions; S or St, starved conditions; B or BafA1, Bafilomycin A1; PI, protease inhibitors (E64d+ pepstatin A). Mean and SD from at least three independent experiments with 10 images assessed per treatment condition. Statistics by two-tailed Student’s *t*-test; *p < 0.05, **p < 0.01.

Since *EPG5* has been reported to function in autophagic and endocytic pathways^12, 20, 24, 25^, we analyzed these functions in patient SFs. Cultured SFs isolated from two patients (patient 1.2 and 4.1) were treated with nutrient-rich or starvation medium for 6 hours with/without the lysosomal inhibitor Bafilomycin A1 (BafA1), then LC3 expression determined by western blot. LC3 is one of the core autophagy genes and a mammalian homologue of yeast Atg8^26^. LC3-I localizes to the cytosol, and is converted into the membrane-bound form LC3-II as autophagy progresses, which specifically localizes on the autophagosome membrane. LC3-II is continuously generated and degraded during the autophagic degradation pathway. The lysososmal inhibitor BafA1 blocks autophagic degradation and causes an accumulation of LC3-II compared with BafA1 non-treated cells. The differences in LC3-II levels in the presence and absence of BafA1 indicated “autophagic flux”, an estimation of the amount of autophagosomes that should have been degraded during that period. Immunoblotting showed a slight accumulation of LC3-II in VICIS SFs under starved conditions without BafA1 (Fig. 2a). Under starved conditions, autophagic flux was reduced in VICIS SFs (Fig. 2a). Immunofluorescence analysis confirmed the accumulation of LC3-containing vacuoles in VICIS SFs under starved conditions (Fig. 2b). These results indicate that the autophagic degradation pathway is slowed in VICIS SFs leading to accumulation of un-degraded autophagic vacuoles within cells.

### Reduced autophagosome-lysosome fusion in VICIS SFs

Next we investigated which process of autophagy is impaired in VICIS. Lack of LC3-I accumulation in VICIS SFs (Fig. 2a) suggested an impairment of autophagosome degradation rather than impairment of an initial step of autophagy, such as the conversion of LC3-I to LC3-II. Immunofluorescence analysis of LC3 (Fig. 2b) supported a defective clearance of autophagic vacuoles. Therefore, we next investigated the fusion of autophagosomes with lysosomes, which results in degradation of cytoplasmic material by the acidic lysosomal environment and hydrolases. In order to directly observe colocalization of autophagosomes and lysosomes, we performed co-immunostaining of LC3 and the lysosomal marker Lamp1 in SFs in the presence of lysosomal PI (protease inhibitor) that prevents lysosomal degradation of LC3 protein. VICIS SFs showed a reduced ratio of colocalization both in nutrient-rich and starved conditions (Fig. 2c), suggesting an impairment of autophagosome-lysosome fusion in VICIS SFs.

### Normal lysosomal function and endocytic degradation in VICIS SFs

It has been previously shown that a KO of mouse *Epg5* causes defective degradation via the endocytic pathway^25^. To assess endocytic degradation in VICIS SFs, we firstly measured the activity of eight lysosomal enzymes (Supplementary Fig. S4a) and processing of cathepsin D (Supplementary Fig. S4b). The results indicated that lysosomal proteolytic activity was normal in VICIS SFs. Next, SFs were treated with DQ-BSA reagent, which is taken up by endocytosis and generates fluorescence after being cleaved within lysosomes. After six hours of treatment there were similar levels of fluorescence in normal and VICIS SFs, indicating comparable activities of endocytic degradation (Supplementary Fig. S4c). Uptake of BSA conjugate was normalized using a second fluorescence (AlexaFluor 488)-conjugated BSA, which does not require cleavage to fluoresce. These results indicated normal endocytic uptake and normal lysosomal degradation in VICIS SFs. We also assessed endocytic degradation using EGFR (epidermal growth factor receptor) dynamics. Upon EGF stimulation, EGF-bound EGFR on the cell surface is internalized by endocytosis. The majority of EGFR is subsequently transported to lysosomes and degraded. A time course of EGFR degradation in VICIS SFs after EGF treatment showed comparable EGFR degradation to EGF-treated normal SFs (Fig. 2d), confirming normal endocytic uptake and lysosomal degradation.

### Defective autophagy in *EPG5* depleted cell lines caused by impaired autophagosome-lysosome fusion

**Figure 3:**
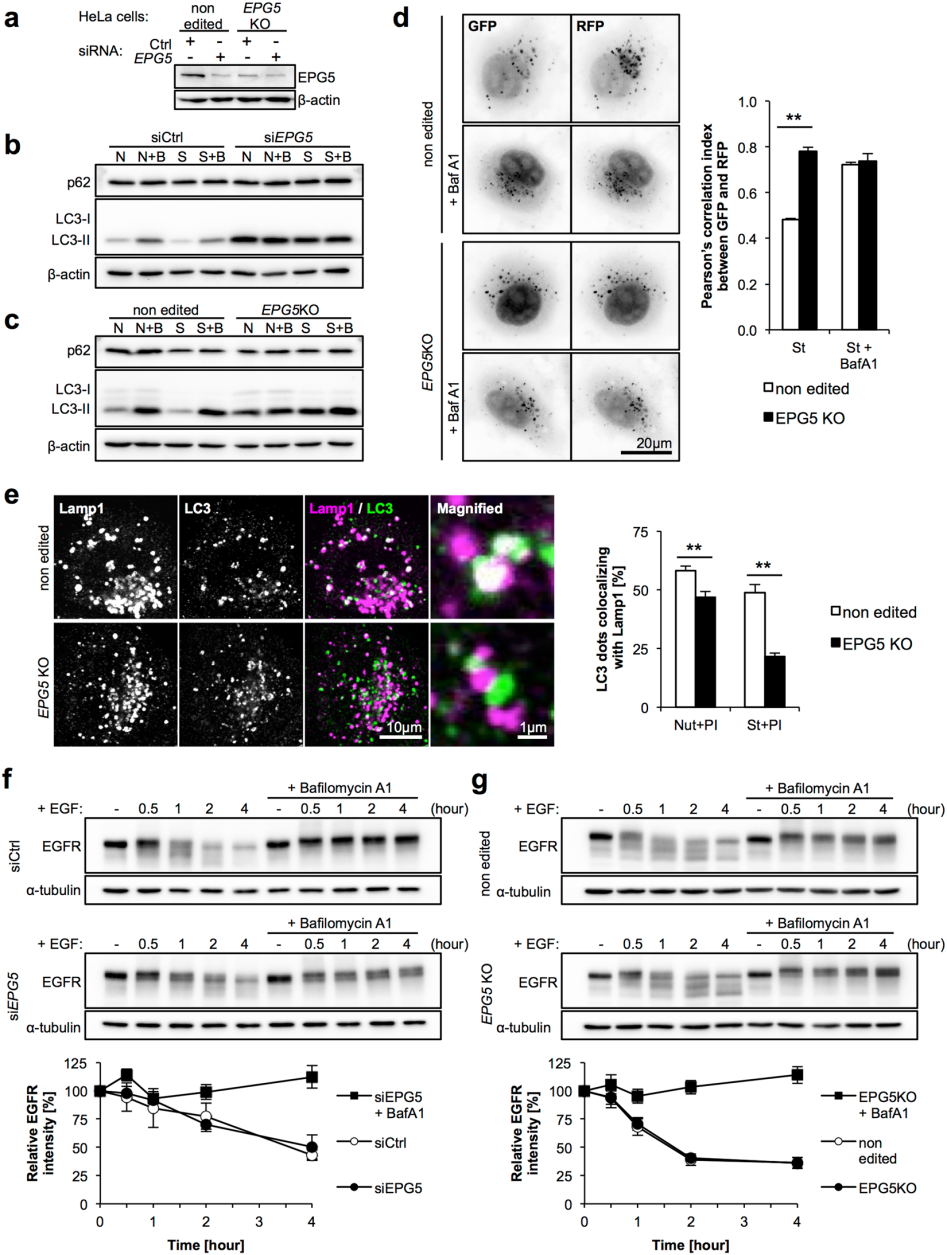
Autophagic impairment is caused by reduced fusion between autophagosomes and lysosomes in *EPG5* KD and KO cells. (**a**) *EPG5* depletion by siRNA treatment. (**b**,**c**) LC3-II has accumulated and autophagic flux is stopped in *EPG5* KD and KO HeLa cells. (**d**) Tandem fluorescent-tagged LC3 (tf-LC3) assay indicated reduced autophagosome-lysosome fusion. *EPG5* KO cells were infected with a retroviral mRFP-GFP-LC3 construct, then incubated in nutrient-rich or starved media with/without BafA1 for two hours. BafA1 inhibited lysosomal V-ATPase and led to disruption of the lysosomal acidic environment, as well as inhibition of autophagosome-lysosome fusion, which we used as the negative control for this experiment. RFP and GFP fluorescence images were obtained, and Pearson’s correlation index was calculated to show colocalization between RFP and GFP. Higher colocalization indicates accumulation of autophagosomes. (**e**) Colocalization of punctate LC3 staining with Lamp1 in the presence of protease inhibitors was lower in *EPG5* KO cells under both nutrient rich and starved conditions. (**f**,**g**) Endocytic degradation of EGFR was normal in *EPG5* KD and KO cells. N or Nut, nutrient-rich conditions; S or St, starved conditions; B or BafA1, Bafilomycin A1; PI, protease inhibitors (E64d+ pepstatin A). The mean and SD are from at least three independent experiments, with 10 images assessed per treatment condition. Statistical analysis was performed using a two-tailed Student’s *t*-test; **p < 0.01.

Since VICIS SFs showed mild cellular phenotypes involving autophagy, we decided to analyze *EPG5* function in *EPG5* depleted cell lines. In HeLa cells, we performed *EPG5* KD using siRNA, and generated an *EPG5* KO cell line using CRISPR/Cas9 system (Fig. 3a). *EPG5* KD and KO cells showed a complete block of autophagic flux (Fig. 3b and c) and accumulation of LC3-II, indicating a fundamental role of *EPG5* in autophagy. Next, we analyzed autophagosome-lysosome fusion in *EPG5* KO cells using two approaches: tandem-fluorescent (RFP and GFP) tagged LC3 (tfLC3) and colocalization of LC3 and Lamp1. The tfLC3 construct (mRFP-GFP-LC3) was firstly transfected into *EPG5* KO cells^27^. As LC3-II localizes on the autophagosomal membrane, both RFP and GFP signals can be detected in autophagosomes. When fused with lysosomes, autophagosomes become autolysosomes and the acidic environment quenches the GFP signal while the RFP signal remains relatively stable. As a result, autolysosomes exhibit an RFP signal only, enabling distinction between autophagosomes and autolysosomes. The tfLC3 assay exhibited an almost complete merge of GFP and RFP signals in *EPG5* KO cells suggesting accumulation of autophagosomes before fusion (Fig. 3d). The ratio of colocalization between LC3 and Lamp1 decreased in *EPG5* KO cells both in nutrient-rich and starved conditions (Fig. 3e). These results clearly indicate that *EPG5* functions in autophagosome-lysosome fusion.

### Normal endocytic degradation in *EPG5* depleted cell lines

To confirm the normal endocytic degradation observed in VICIS SFs, we performed the same assay for EGFR degradation in *EPG5* KD and KO cells. Despite *EPG5* KD and KO cells demonstrating clear defects in autophagic function, endocytic function, as measured by EGFR degradation, was completely normal (Fig. 3f and g), indicating *EPG5* is not involved in endocytosis.

## Discussion

We have identified nine patients from seven families with genetically confirmed VICIS. ACC, cataracts, hypopigmentation, cardiomyopathy, and immune dysfunction have been indicated as the five principle features of VICIS^1^. In our study, all patients had ACC and recurrent infections, seven patients had hypopigmentation, three patients had cardiac involvement, and no patients had cataracts. Thus, the proposed principle features of VICIS were not consistently present in our patient. Two Japanese VICIS patients previously reported also had no cataracts^4^. These findings suggest that the frequency of cataracts may be affected by race. Recently Byrne *et al*.^18^ proposed that profound developmental delay, progressive microcephaly, and a failure to thrive, are equally consistent features of VICIS. In our cohort, all patients demonstrated profound developmental delay, while progressive microcephaly and failure to thrive were exhibited in most patients. Thus, our findings support those of Byrne *et al*.^18^. Moreover, we propose three additional features of VICIS; epilepsy, high-arched palate and elevated AST/ALT, based upon their presence in all or most of our patients. MRI findings in our study were basically comparable to those in previous reports^18^. Complete ACC and pontine hypoplasia were consistently present while paucity of white matter, irregularity of the ventricular wall, ventricular dilation, and cerebral atrophy were present in most patients. It is of note that Probst bundle was not detected in any patients. It may reflect a failure to form commissural axons^28^. Previously, it was reported that muscle biopsies in patients with VICIS were characterized by increased variability in fiber size, increase in internal and centralized nuclei, abnormal glycogen accumulation on light microscopy^6, 7, 12, 18^. There might be some histological overlap between VICIS and a number of primary neuromuscular disorders where aberrant autophagy was implicated as important secondary mechanisms, including vacuolar myopathies, centronuclear myopathies and glycogen storage disorders^29–31^. In our study, muscle biopsies were non-specific and not diagnostic, indicating that a negative result did not exclude a VICIS diagnosis. Because the precise mechanism for the abnormal muscle histopathologies remains to be clarified, further investigation should be needed. In conclusion, VICIS should be suspected in any child with ACC and profound developmental delay, even if some of the principle features of VICIS are absent. It should be noted that while the type of complications in other organs differed between patients, the severity of developmental delay was uniformly profound and indicated a consistently severe dysfunction of brain development in our patients.

The majority of mutations identified in this study produced truncations and/or caused profound dysfunction. Five nonsense mutations, two frameshift mutation﻿s, and one multi-exon deletion are likely to produce a non-functional protein, and are thus essentially null alleles. Two of the three splicing mutations were assessed by RT-PCR to locate mutations leading to in-frame deletions of 66 and 15 amino acid residues, respectively, which could potentially cause significant functional defects. The canonical +1 splicing mutation (c.6766 + 1 G > C) was not assessed due to lack of RNA. Nonetheless, it is likely to cause aberrant splicing that induces major functional defects in the translated protein. It is of considerable interest that two *EPG5* variants resided in the initial start codon. Since these mutations are likely to alter the translation start site, mutated *EPG5* protein would have a truncated N-terminal structure, resulting in substantial functional defects. One missense mutation (c.3044 C > T) was predicted to be pathogenic. A recent report by Kane *et al*.^32^ demonstrated aberrant splicing was induced by a missense mutation c.1007 A > G in *EPG5*, however we were not able to test if this occurred with c.3044 C > T because RNA was not available.

Previous reports have demonstrated that most *EPG5* mutations identified in VICIS appear to be null alleles^18^. Very few missense mutations have been identified^18^, and of these, some may induce aberrant splicing. Furthermore, *Epg5* KO mice are viable and show an overlapping phenotype with VICIS patients, although with some distinct features^24^. This suggests VICIS pathogenesis is likely driven by a significant functional or even complete defect in *EPG5*.

The two patients for whom we analyzed SFs (patient 1.2 and 4.1) exhibited typical clinical VICIS symptoms including ACC, profound developmental delay and early onset seizure, with one patient also showing cardiomyopathy. Genetically, these patients harbored variants in the initiation codon and frameshift mutation (patient 1.2 and 4.1). Functional studies using their SFs however, demonstrated only a partial defect in autophagy compared to *EPG5* KD and KO cells. The discrepancy between clinical severity and cellular phenotype may arise from differences in cell types or, in this case, from retained function of the truncated *EPG5* protein induced by variants in the initial codon. Compound heterozygous mutations of null alleles would most likely cause more profound functional defects in SFs, however we were not able to test this hypothesis. Therefore, further studies are needed to elucidate the mechanisms underlying the neurodevelopmental features in VICIS, which are possibly common to other congenital neuronal disorders associated with autophagy^33^.

In this study, we demonstrated defects in autophagosome-lysosome fusion in VICIS SFs and *EPG5* depleted cells. Several mechanisms have been proposed for *EPG5* function. Consistent with our findings, the work that first described an association between VICIS and *EPG5* observed an accumulation of autophagosomes, indicative of impaired autophagosome-lysosome fusion in VICIS^12^. Another group reported *EPG5* functions in autophagosome maturation, and endocytic degradation and recycling^20, 24, 25^ where loss of *EPG5*/*Epg5* impaired autolysosomal proteolysis activity, and delayed endosomal trafficking. The same group recently showed *EPG5* functions as a tethering factor that facilitates the fusion between endosomes/lysosomes and autophagosomes^34^. This is supported by our data where autophagy was significantly impaired in *EPG5* KD and KO cells, yet endocytic degradation remained normal. Thus, we propose the function of *EPG5* is limited to autophagosome-lysosome fusion.

In conclusion, we have identified novel, compound heterozygous mutations in patients with VICIS, and demonstrated partial impairment of autophagosome-lysosome fusion in VICIS SFs, as well as complete impairment in *EPG5* KD and KO cells. Our study indicates that precise regulation of autophagy is crucial for neuronal development, as well as the normal function of other tissues.

## Materials and Methods

### Patients

We reviewed the clinicopathological features of nine affected patients (three male/six female) from seven families with genetically confirmed VICIS. Eight patients were not clinically diagnosed with VICIS but were categorized as having undiagnosed neurological disorders and DNA of parent-patient trios subjected to whole exome sequencing. One patient (7.1) was clinically diagnosed with VICIS, and their DNA was subjected to targeted gene panel sequencing analysis for *EPG5*. This study was approved by the institutional review board in Nagoya City University Graduate School of Medical Sciences (#169), and were carried out in accordance with the approved guidelines. Patients’ parents provided written informed consent for genetic analysis and for the usage of recognizable clinical photographs for the online open-access publication.

### Sequence analyses

Whole exome sequencing was performed on eight parent-patient trios (families 1–6). Genomic DNA was captured using the SureSelect XT Human All Exon V5 capture library (Agilent Technologies, Santa Clara, CA, USA), and sequenced using the Illumina HiSeq 2000 (Illumina, San Diego, CA, USA) with 100 bp paired-end reads. Exome data processing, variant calling and variant annotation were performed as described previously^35, 36^. Multiplex targeted sequencing analysis was performed on one additional patient (patient 7.1). Amplicon libraries corresponding to the 44 exons of the *EPG5* gene were prepared with an Ion AmpliSeq Custom Panel (Thermo Fisher Scientific, Waltham, MA, USA), and sequenced with an Ion Torrent Personal Genome Machine (PGM) system^37^. Sequence data was analyzed using a CLC Genomics Workbench 7.0 (CLC bio, Aarhus, Denmark) and Ion reporter (Thermo Fisher Scientific). Identified mutations were validated in all nine patients by Sanger sequencing. The breakpoints in patient 7.1 were PCR amplified and sequenced. The ClinVar accession numbers for the DNA variants reported in this paper are SCV000328413, SCV000328414, SCV000328415, SCV000328416, SCV000328417, SCV000328418, and SCV000328419 (ClinVar, https://www.ncbi.nlm.nih.gov/clinvar/).

### Muscle biopsy

Muscle biopsy was performed in four patients. Biopsy specimens were either frozen in liquid nitrogen-cooled isopentane for histochemistry or fixed with glutaraldehyde for electron microscopy. Transverse serial frozen sections of 10-μm thickness were stained with hematoxylin and eosin, modified Gomori trichrome, and a battery of histochemical methods. For electron microscopy, biopsy specimens were fixed in buffered 2% isotonic glutaraldehyde at pH 7.4, post-fixed in osmium tetroxide, and embedded in epoxide resin. Ultrathin sections were stained with uranyl acetate and lead nitrate and examined with an H-700 electron microscope (Hitachi, Tokyo, Japan).

### Cell culture and generation of *EPG5* KD and KO lines

Cultured SFs from two affected individuals (patient 1.2 and 4.1) were established by conventional methods^38^. Representative data from patient 4.1 SF are shown in the figures, with comparable phenotypes observed in SFs from patient 1.2. Normal SFs were purchased (Gibco, Waltham, MA, USA, Lonza, Basel, Switzerland, and Kurabo, Osaka, Japan). For the KD of *EPG5*, we transfected *EPG5* siRNA^20^ with Lipofectamine RNAiMax (Thermo Fisher Scientific) into HeLa cells for 96 hours (transfected twice, every 48 hours). *EPG5* KO cells were generated with HeLa cells by cloning guide RNA within the exon 13 of *EPG5* (designed using CRISPR design tool, http://crispr.mit.edu) into the pSpCas9(BB)-2A-GFP (PX458) vector (Addgene, Cambridge, Massachusetts, USA) that uses a hU6 promoter. We transfected the cloned vector into HeLa cells for 48 hours, then GFP expressing cells collected by fluorescence activated cell sorting (FACS). Single clones were isolated by diluting the cell suspension and culturing in 96-well plates. Genomic modification of *EPG5* was confirmed with Sanger sequencing. In this study we used a clone with homozygous mutation of c.2512_2515delCTTC (p.Leu838Trpfs*34). Cells were cultured and maintained in complete medium (Dulbecco’s Modified Eagle Medium, DMEM) supplemented with 10% fetal bovine serum (FBS).

### Assays for autophagy and endocytosis

For the nutrient-rich and starved conditions, cells were incubated with complete medium or Earle’s Balanced Salt Solution (EBSS), respectively, following two phosphate-buffered saline (PBS) washes. To measure autophagosome-lysosome fusion, the tandem fluorescent-tagged LC3 (tfLC3) construct (described previously^27^) was transfected into cells 48 hours prior to measuring GFP and RFP fluorescence. Specific activities of lysosomal enzymes were measured with total cell lysates using artificial 4-Methylumbelliferone (4-MU) fluorescence substrates^39^. Sonicated cell lysates were incubated with each of the artificial 4-MU substrates (M1633, M2133, M3633, M3567, M8527, M9766, M7633, M9130; Sigma-Aldrich, St. Louis, Missouri, USA) in acidic acetate buffer or citrate-phosphate buffer for 3 hours at 37 °C and the reaction stopped by adding glycine-NaOH buffer (0.2 M Glycine, 0.2 M NaOH). Fluorescence at Em 450 nm/Ex 365 nm was measured with a microplate reader (Corona, Hitachinaka, Ibaraki, Japan). The standard, 4-MU, (M1381; Sigma-Aldrich) was also measured in the same buffer, and enzyme activities were calculated as nmol/protein mg/h. To assess endocytic activity, DQ-Red BSA and AlexaFluor488-BSA (Invitrogen, Waltham, Massachusetts, USA) were incubated in culture medium at 50 μg/ml for 6 hours. Cells were fixed and fluorescence intensity was quantified with fluorescence microscope and ImageJ software (NIH). For the EGFR degradation assay, cultured cells were washed twice with warm serum-free DMEM, and pre-incubated in serum-free DMEM for two hours before adding EGF. BafA1 (bafilomycin A1) was added 1 hour before EGF. EGF (53003018, Thermo Fisher Scientific) was added to the medium (100 ng/ml) at time 0, and cells were washed once with PBS and collected at the indicated time points. Samples were subsequently lysed and analyzed by immunoblotting for EGFR and α-tubulin.

### Antibodies and reagents

Primary antibodies for western blotting (WB) and immunocytochemistry (ICC) were purchased and used at the indicated dilution; anti-LC3 (PM036, MBL; WB 1:3000; ICC 1:1000), anti-p62 (PM045, MBL; WB 1:5000), anti-Lamp1 (clone H4A3, sc-20011, Santa Cruz Biotechnology; ICC 1:1000), anti-EGFR (MI-12-1, MBL; WB 1:500), anti-cathepsin D (sc-6486, Santa Cruz Biotechnology; WB 1:1000), anti-EPG5 (sc-85198, Santa Cruz Biotechnology; WB 1:200), anti-GAPDH (MAb314, Chemicon; WB 1:5000), anti-α-tubulin (clone B-5-1-2, T5168, Sigma-Aldrich; WB 1:5000), anti-β-actin (clone 6D1, M177-3, MBL; WB 1:3000). BafA1 was used at 125 nM. PI comprised 10 μg/ml E64d and 10 μg/ml pepstatin A.

### Western blotting

Cells were lysed directly by sodium dodecyl sulphate (SDS) sample buffer and subjected to SDS-PAGE, followed by transfer onto PVDF membrane (Millipore, Billerica, Massachusetts, USA). The membrane was blocked with 1% skim milk (Nacalai Tesque, Kyoto, Japan) in TBST (0.1% Tween-20 in Tris-buffered saline) for 20 min at room temperature, and incubated with a primary antibody as described above in 1% skim milk TBST for 1 hour at room temperature. For EPG5 immunoblotting, the primary antibody was incubated at 4 °C overnight. The membrane was washed with TBST for at least 30 min and incubated with HRP-conjugated secondary antibody (GE Healthcare, Chicago, Illinois, USA) at room temperature for 1 hour. After the final wash with TBST, the membrane was treated with Luminata Forte western HRP substrate (GE Healthcare) and chemiluminescence was visualized with an analyzer (ChemiDoc Touch; BioRad, Hercules, California, USA). Band intensities were measured using ImageJ software.

### Immunocytochemistry

Cells were grown on glass cover slips in culture dishes, treated, and fixed with 4% paraformaldehyde in PBS for 20 min at room temperature. Cells were then permeabilized with 50 μg/ml digitonin in PBS for 10 min at room temperature, and immunostained with primary antibodies in 0.2% gelatin PBS for 1 hour at room temperature. After washing cells with PBS, they were incubated with fluorescence-conjugated secondary antibodies for 1 hour at room temperature together with DAPI for nuclear staining. Images were acquired with a wide field fluorescence microscope (IX83; Olympus, Tokyo, Japan) or with a confocal laser-scanning microscope (FV1000, Olympus), and analyzed using ImageJ software.

## Electronic supplementary material

supplement
