# Supplementary material for: Defects in autophagosome-lysosome fusion underlie Vici syndrome, a neurodevelopmental disorder with multisystem involvement
Source: Sci Rep. 2017 Jun 14;7:3552. doi: 10.1038/s41598-017-02840-8 (PMC5471274; doi:10.1038/s41598-017-02840-8)
Supplement: Supplementary file 1 — supplement [file 41598_2017_2840_MOESM1_ESM.pdf]

Defects in autophagosome-lysosome fusion underlie Vici syndrome, a neurodevelopmental disorder with multisystem involvement

Ikumi Hori[1, +], Takanobu Otomo[2, 3, +], Mitsuko Nakashima[4], Fuyuki Miya[5, 6], Yutaka Negishi[1], Hideaki Shiraishi[7], Yutaka Nonoda[8], Shinichi Magara[9], Jun Tohyama[9], Nobuhiko Okamoto[10], Takeshi Kumagai[11], Konomi Shimoda[12], Yoshiya Yukitake[13], Daigo Kajikawa[14], Tomohiro Morio[15], Ayako Hattori[1], Motoo Nakagawa[16], Naoki Ando[1], Ichizo Nishino[17], Mitsuhiro Kato[18], Tatsuhiko Tsunoda[5, 6], Hirotomo Saitsu[4, 19], Yonehiro Kanemura[20, 21], Mami Yamasaki[22], Kenjiro Kosaki[23], Naomichi Matsumoto[4], Tamotsu Yoshimori[2, 3], Shinji Saitoh[1, *]

[1]Department of Pediatrics and Neonatology, Nagoya City University Graduate School of Medical Sciences, Nagoya 467-8601, Japan.
[2]Department of Genetics, Osaka University Graduate School of Medicine, Osaka 565-0871, Japan.
[3]Research Center for Autophagy, Osaka University Graduate School of Medicine, Osaka 565-0871, Japan.
[4]Department of Human Genetics, Yokohama City University Graduate School of Medicine, Yokohama 236-0004, Japan.
[5]Department of Medical Science Mathematics, Medical Research Institute, Tokyo Medical and Dental University, Tokyo 113-8510, Japan.
[6]Laboratory for Medical Science Mathematics, RIKEN Center for Integrative Medical Sciences, Yokohama 230-0045, Japan.
[7]Department of Pediatrics, Hokkaido University Graduate School of Medicine, Sapporo 060-8638, Japan.
[8]Department of Pediatrics, Kitasato University School of Medicine, Sagamihara 252-0373, Japan.
[9]Department of Pediatrics, Epilepsy Center, Nishi-Niigata Chuo National Hospital, Niigata 950-2085, Japan.
[10]Department of Medical Genetics, Osaka Medical Center and Research Institute for Maternal and Child Health, Osaka 594-1101, Japan.
[11]Department of Pediatrics, Wakayama Medical University, Wakayama 641-8509, Japan.
[12]Department of Pediatrics, Graduate School of Medicine, The University of Tokyo, Tokyo 113-8655, Japan.
[13]Department of Neonatology, Ibaraki Children’s Hospital, Mito 311-4145, Japan.
[14]Department of Child Health, Faculty of Medicine, Tsukuba University, Tsukuba 305-8576, Japan.
[15]Department of Pediatrics, Faculty of Medicine, Tokyo Medical and Dental University, Tokyo 113-8519, Japan.
[16]Department of Radiology, Nagoya City University Graduate School of Medical Sciences, Nagoya 467-8601, Japan.
[17]Department of Neuromuscular Research, National Institute of Neuroscience, National Center of Neurology and Psychiatry, Tokyo 187-8551, Japan.
[18]Department of Pediatrics, Showa University School of Medicine, Tokyo 142-8666, Japan.
[19]Department of Biochemistry, Hamamatsu University School of Medicine, Hamamatsu 431-3192, Japan.
[20]Division of Regenerative Medicine, Institute for Clinical Research, Osaka National Hospital, National Hospital Organization, Osaka 540-0006, Japan.
[21]Department of Neurosurgery, Osaka National Hospital, National Hospital Organization, Osaka 540-0006, Japan.
[22]Department of Neurosurgery, Takatsuki General Hospital, Osaka 569-1192, Japan.

[23]Center for Medical Genetics, Keio University School of Medicine, Tokyo 160-8582, Japan.

*Corresponding author: S.S., ss11@med.nagoya-cu.ac.jp

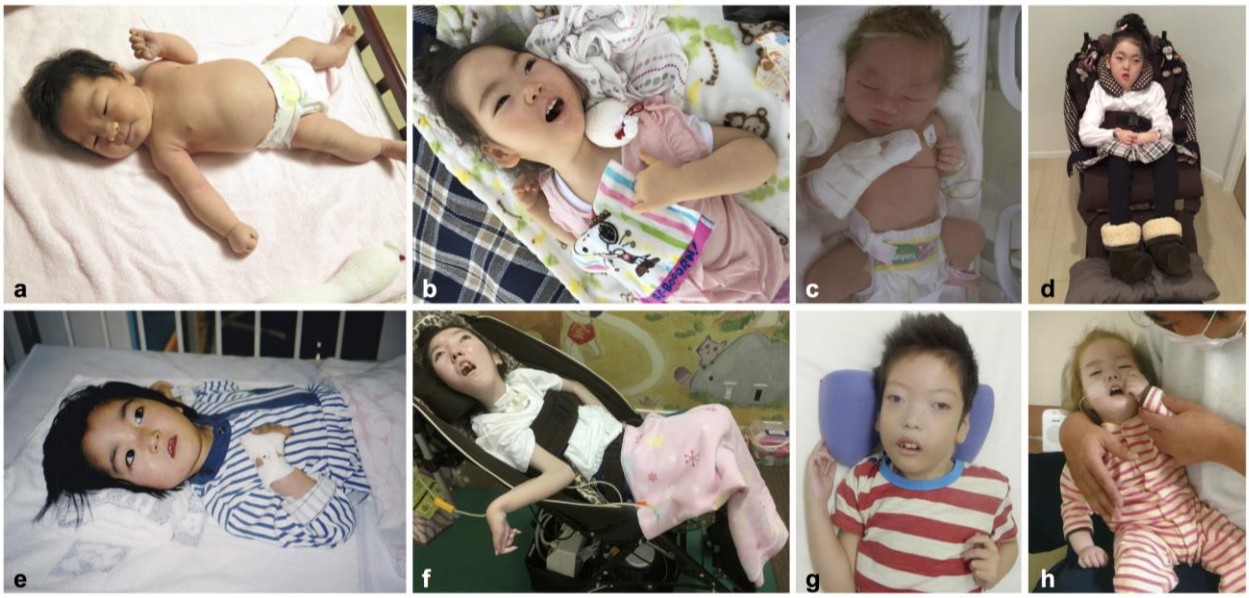

Figure S1. Clinical photographs. Patient 1.1 (a, b), patient 1.2 (c, d), patient 2.1 (e), patient 2.2 (f), patient 4.1 (g), and patient 5.1 (h) at different ages. Most patients have hypopigmentation, upslanted palpable fissures and telecanthus.

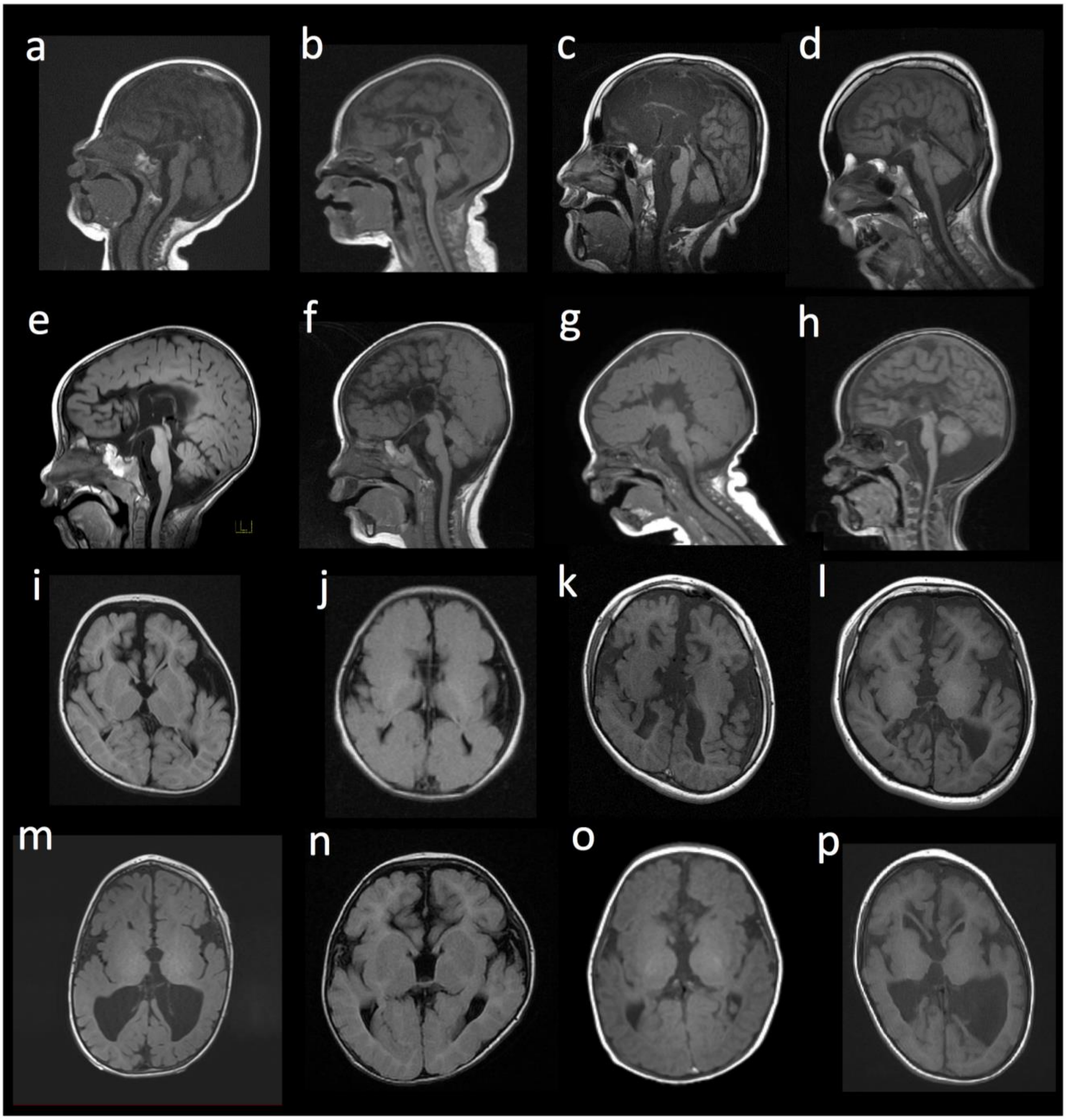

Figure S2. Brain MRI scans of patients. Representative T1-weighted midsagittal and axial images from patient 1.1 at age 5 months (a, i), patient 1.2 at age 4 days (b, j), patient 2.1 at age 10 years (c, k), patient 2.2 at age 13 years (d, l), patient 3.1 at age 1 year (e, m), patient 4.1 at age 2 years (f, n), patient 5.1 at age 3 months (g, o), and patient 6.1 at age 3 years (h, p). All patients exhibited complete ACC and additional CNS abnormalities including paucity of white matter, irregularity of the ventricular wall, ventricular dilation, delayed myelination, pontine hypoplasia, cerebellar hypoplasia, and cerebral atrophy.

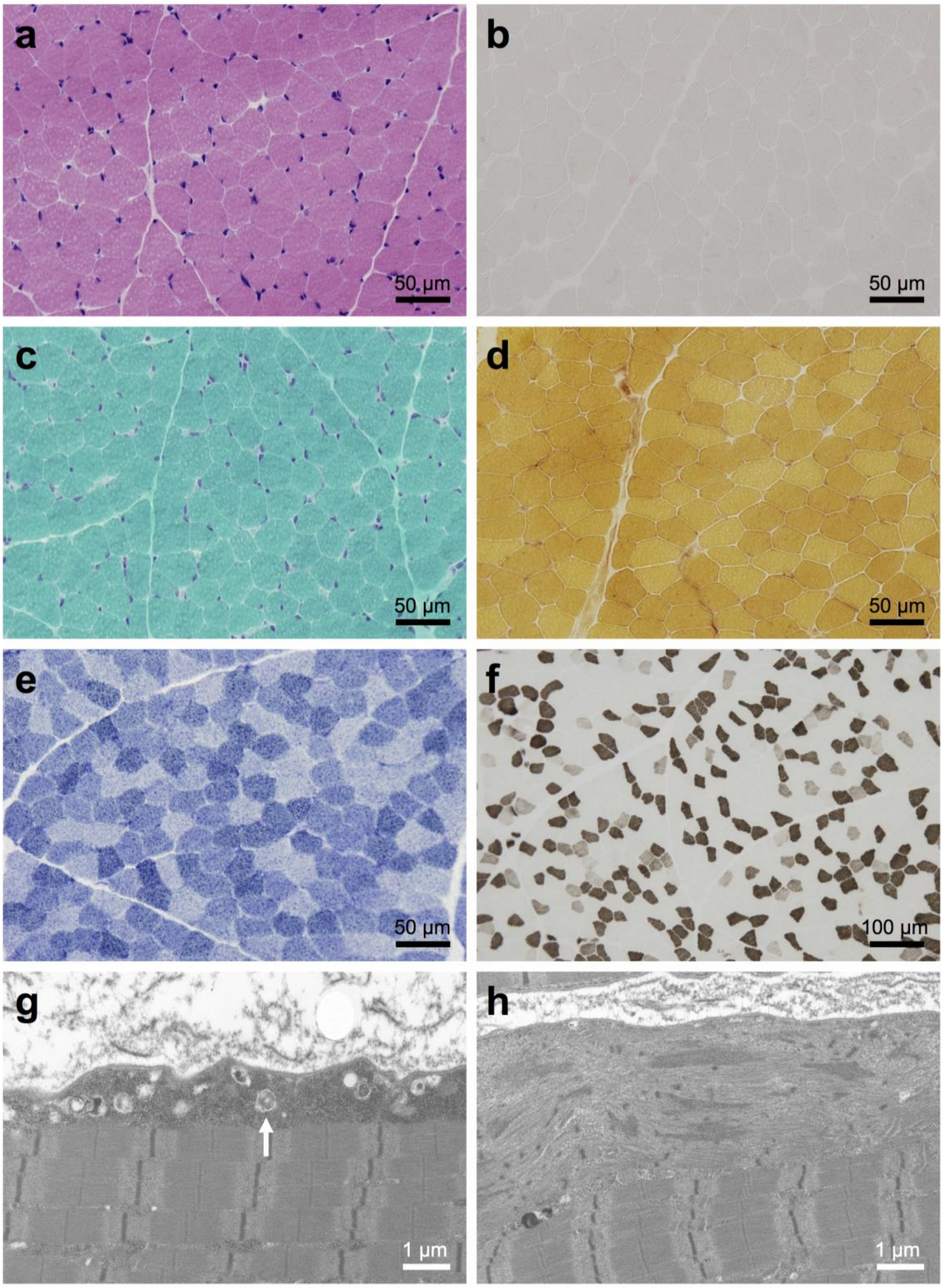

Figure S3. Histological and ultrastructural findings in muscle biopsies from patient 1.1 (a–f) and patient 2.1 (g, h). Hematoxylin and eosin staining (a), acid phosphatase activity (b), modified Gomori trichrome staining (c), non-specific esterase activity (d), staining for NADH-tetrazolium reductase activity (e), and staining for myofibrillar actomysin ATPase activity after pre-incubated at pH 10.6 (f), showed fiber-type disproportion with type 2 atrophy. Electron microscopy demonstrated only slight abnormalities of autophagosome vacuoles (arrow) (g, h).

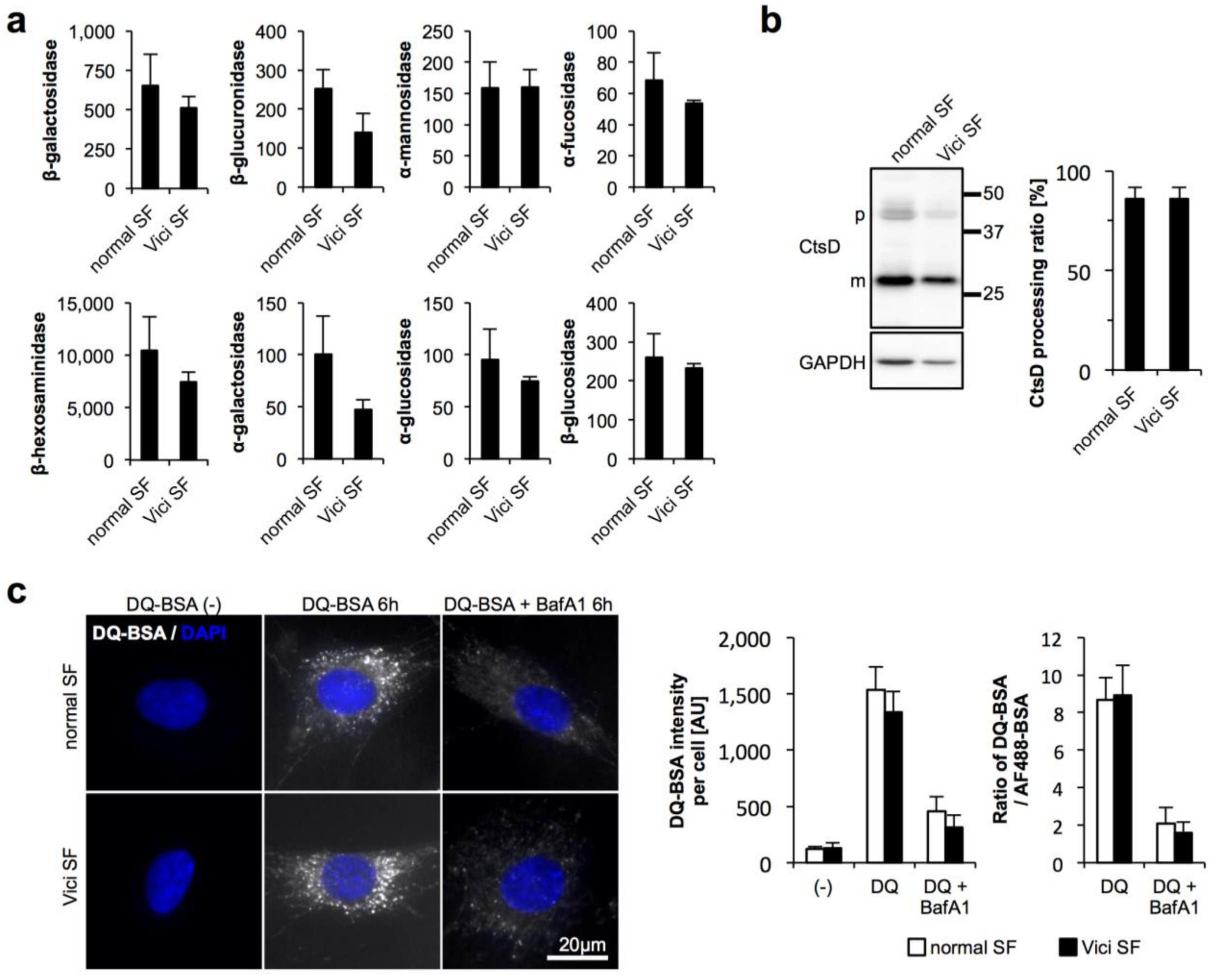

Figure S4. Normal endocytic degradation in Vici syndrome SFs. (a) The specific activity of lysosomal enzymes were comparable in normal and Vici syndrome SFs. Enzyme activities are shown as nmol/protein mg/h. (b) Cathepsin D (CtsD) processing was normal in Vici syndrome SFs. p, premature form; m, mature form. Processing rate was calculated from band intensities as "mature form / (premature form + mature form) x 100". (c) Uptake and proteolytic cleavage of endocytosed material was measured using DQ-BSA. Cells were incubated with DQ-BSA and AlexaFluor(AF)488-BSA for 6 hours with/without BafA1 and fluorescence was observed. Both fluor-conjugated BSA are taken up by endocytosis and transported to lysosomes. DQ fluorescence is generated upon proteolytic processing of DQ-BSA, and BSA uptake was evaluated by AF488 fluorescence. Both DQ and AF488 intensities were comparable between normal and Vici syndrome SFs, and treatment with the lysosomal inhibitor BafA1 reduced DQ fluorescence. Mean and SD from three independent experiments are shown.

Table S1. Clinical features of nine patients with Vici syndrome

Family	1		2		3	4	5	6	7
Subject	1.1	1.2	2.1	2.2	3.1	4.1	5.1	6.1	7.1
Gestational age [week-day]	40w0d	40w1d	39w0d	37w3d	37w0d	38w0d	38w2d	37w0d	40w4d
Birth weight [g]	3214	3305	2600	2624	2495	3026	2831	2238	2850
(SD)	(+0.5)	(+0.8)	(-1.0)	(-0.9)	(-1.2)	(+0.1)	(-0.4)	(-1.9)	(-0.4)
Birth length [cm]	50.5	48.0	49.0	47.8	47.0	49.0	48.0	44.6	49.0
(SD)	(1.0)	(-0.2)	(0)	(-0.3)	(-1.0)	(0)	(-0.5)	(-1.8)	(+0.3)
Birth OFC [cm]	33.0	32.0	31.0	32.3	33.5	34.0	32.0	31.0	31.0
(SD)	(-0.1)	(-0.9)	(-1.8)	(-0.6)	(0.0)	(+0.4)	(-1.1)	(-1.6)	(-1.6)
Last follow-up age [year-month]	7y5m	2y6m	14y4m	15y2m	4y5m	6y3m	1y9m	3y3m	2y10m
Last weight [kg]	22.4	11.8	16.4	19.7	10.7	13.7	7.3	11.0	10.6
(SD)	(-0.3)	(-0.3)	(-3.7)	(-3.9)	(-2.7)	(-2.1)	(-3.3)	(-1.5)	(-1.5)
Last length [cm]	130.3	96.5	126.0	122.7	94.1	103.0	76.9	93.8	90.0
(SD)	(+1.8)	(+2.6)	(-5.8)	(-6.6)	(-2.2)	(-2.4)	(-2.1)	(-0.1)	(-0.3)
Last OFC [cm]	48.0	47.0	45.8*	50.8	46.0	47.5	43.5	47.0	42.5
(SD)	(-2.6)	(-0.6)	(-1.6)	(-2.7)	(-2.9)	(-2.5)	(-2.6)	(-0.9)	(-3.4)

OFC, occipitofrontal head circumference.

*The last OFC of patient 2.1 was evaluated at 2 years and 3 months.
